# Assessing the Acceptability of Home Blood Monitoring for Patients With Cancer Who Are Receiving Systemic Anticancer Therapy From a Patient, Caregiver, and Clinician Perspective: Focus Group and Interview Study

**DOI:** 10.2196/39815

**Published:** 2023-01-06

**Authors:** Amy Vercell, Sally Taylor, Janelle Yorke, Dawn Dowding

**Affiliations:** 1 The Christie NHS Foundation Trust Manchester United Kingdom; 2 Division of Nursing, Midwifery and Social Work, School of Health Sciences Faculty of Biology, Medicine and Health The University of Manchester Manchester United Kingdom; 3 National Institute for Health and Care Research Applied Research Collaboration Greater Manchester Greater Manchester United Kingdom

**Keywords:** chemotherapy, digital technology, digital nursing, nurse-led care, oncology, point-of-care systems, self-management, self-testing, systemic anticancer therapy, telemedicine, mobile phone

## Abstract

**Background:**

Regular blood testing is an integral part of systemic anticancer therapy delivery. Blood tests are required before every administration of treatment to ensure that a patient is sufficiently well to receive it. Blood testing is burdensome for patients as they require either an extra visit within 48 hours of planned administration of treatment or a significantly long visit if performed on the day of treatment. The additional time for appointments can have a significant impact on the quality of life of someone who is living with cancer. In the United Kingdom, the COVID-19 pandemic created unprecedented disruption to the delivery of cancer care. Face-to-face hospital visits were reduced, resulting in the need to develop more innovative ways of working to minimize treatment interruptions. This led to significant uptake of digital technologies, with new models of care rapidly deployed across the UK health service to meet these challenges.

**Objective:**

This study aimed to explore the acceptability of a point-of-care home blood monitoring device for people with cancer who are receiving systemic anticancer therapy, which is being developed in response to the increased need for remote care for patients with cancer.

**Methods:**

Qualitative focus groups and semistructured interviews were conducted with patients (23/47, 49%), caregivers (6/47, 13%), and health care professionals (18/47, 38%) over a 19-month time frame from May 2019 to December 2020. Data were analyzed using framework analysis guided by the Unified Theory of Acceptance and Use of Technology model.

**Results:**

Analysis identified 4 overarching themes: performance expectancy, effort expectancy, social influence, and facilitating conditions.

**Conclusions:**

This study found that patients with cancer, their caregivers, and health care professionals had positive perceptions about home blood monitoring. Although they are often considered synonymously, self-testing and self-management are not mutually exclusive, and this study illustrated some disparity in opinions regarding patient self-management. Home blood monitoring has the potential to provide patients with cancer with a convenient option for blood monitoring. It would minimize hospital attendances, decrease late treatment deferrals, and provide prompt recognition of cancer treatment toxicities, thus enhancing the existing nurse-led protocols and clinical pathways. Home blood monitoring would create a long-term sustainable transformation for the delivery of cancer care, using digital health to act as a facilitator to address a pertinent issue regarding improving the efficiency of hospital resources and increasing the delivery of personalized patient care. Further studies are needed to determine how and where home blood monitoring would fit within clinical pathways, in a way that is robust and equitable.

## Introduction

### Overview

Cancer incidence rates in the United Kingdom are projected to rise by 42.5% over the next 20 years [1], with 50% of the population expected to receive cancer diagnosis [2]. Cancer represents a major health issue and economic burden on health care systems worldwide. Maximizing the efficiency of treatment pathways and optimizing patient outcomes are key priorities [3], with the COVID-19 pandemic greatly emphasizing this need. Approximately 28% of people who have been diagnosed with cancer receive systemic anticancer therapy (SACT) [4]. SACT refers to the systemic delivery of drugs that have antineoplastic effects [5]. These drugs include traditional cytotoxic chemotherapy and new, biological agents such as monoclonal antibodies, targeted therapies, and immunotherapy [6]. Patients receiving SACT experience treatment toxicities, which they must navigate and manage, with support from their caregivers and clinical team. Toxicities can vary in their severity and required management, ranging from supportive medication in an outpatient setting to hospitalization and dose interruptions (which may also include dose reductions) [7]. Before every administration of SACT, the patient undergoes a clinical assessment and full blood screening to ensure that it is safe to administer treatment. This blood test will incur either an additional visit within 48 hours of their intended SACT appointment or can be performed on the day of treatment administration, resulting in a long day for the patient. Either option is burdensome to the patient [8], and during the pandemic, these additional or longer hospital visits lead to increased potential risk of greater transmission of SARS-CoV-2 and increased footfall at the hospital. Neutropenia, thrombocytopenia, and anemia are common reasons for SACT deferral. They often do not directly cause the patient to be acutely unwell, and therefore, related blood levels are the first indication that the treatment cannot proceed.

A recent service evaluation conducted at a UK cancer center explored the incidence of last-minute deferrals owing to neutropenia; 7% (n=224) and 5% (n=169) of patients had their SACT rescheduled owing to neutropenia in the last quarters of 2019 and 2020, respectively [9]. This has a negative impact on the patient, as many patients were told that the treatment could not be administered after they had arrived at the hospital, and on the efficiency of hospital resources. There is anecdotal evidence suggesting that this is a worldwide problem; however, published data are limited. The creation of a point-of-care device that allows the patient to self-test a capillary sample at home to provide a full blood count (FBC) could revolutionize existing clinical pathways. At a time when the pandemic continues to affect care delivery, using innovative technologies can minimize treatment interruptions while promoting remote and ambulatory care. The point-of-care device will require a patient to perform a finger prick test in their own home, with the obtained sample subsequently being analyzed. The FBC will provide a value for total white blood cells, neutrophils, hemoglobin, and platelets, comparable with a venous FBC result analyzed in a hospital laboratory. This will enable clinicians to ascertain whether the patient’s FBC is within the desired parameters to proceed with treatment, without the patient leaving their home. Patients whose FBC is not within the required range for treatment can be assessed remotely. If the patient is well, their treatment can be deferred accordingly. If this remote assessment highlights anything of concern, a proactive review can be arranged to ascertain whether any intervention is required. Prompt detection of neutropenia and monitoring of recovery could positively affect patients’ quality of life and potentially individualize SACT delivery [8].

### Background

The increased burden that an aging population has on our health service has instigated the need for new patient-centered models of care, with the patient being considered as an expert in their care, rather than a passive recipient [10]. Remote monitoring of patients using technology is becoming more prevalent, as is patient self-management, with patient education being a key facilitator to achieve high-quality, safe patient care [11]. A critical appraisal of peer-reviewed papers that explored the impact of mobile health apps for people with cancer who were receiving SACT found statistically significant differences in patient-reported outcomes collected remotely through a smartphone app or internet portal compared with those collected through usual care. These findings illustrated improved symptom control and, thus, quality of life, through remote monitoring [12]. Studies have developed self-management interventions for anticoagulation therapy, asthma, and diabetes. Results illustrated that when patients are trained in self-testing and self-management, anticoagulation therapy is improved [11], acute care interventions for asthma are reduced [13], and blood glucose levels and lifestyle are improved in a more sustainable manner [14]. Characteristics of successful patient self-management interventions include being embedded in clinical pathways and tailored to specific conditions and incorporating regular reviews with health care professionals (HCPs), educational reinforcement, and use of technology [15].

Before the COVID-19 pandemic, delivery of remote outpatient cancer care was being explored, but its progression was impeded by system inertia and slow speed of adoption. This appeared to be owing to several factors, including accreditation; reimbursement; and overall apparent reticence from patients, clinicians, and organizations, combined with a potentially immature digital health infrastructure [16]. The COVID-19 pandemic accelerated the digital health agenda, with many digital health tools moving from being perceived as a potential opportunity to becoming an absolute necessity, thus expediting developments and rapidly increasing uptake [17]. Consequently, remote monitoring and remote consultations have become the norm within oncology care, with benefits being seen in terms of improved patient access and convenience, while facilitating caregiver involvement and maintaining the delivery of clinical services [18].

The urgent and immediate necessity of moving most face-to-face care to remote and remote consultations because of the pandemic has meant that there has been little opportunity for evaluation of its efficacy. This was highlighted in a systematic scoping review of artificial intelligence, telehealth, and related technologies implemented during the pandemic, which identified that the extent of successful real-world applications of these technologies is unclear [19]. This emphasizes the need to ensure that the development of home blood monitoring is implemented with stakeholder involvement, so that it is accepted as a service development by those it will involve, while ensuring that it is fit for the purpose. This is supported by the Integrate, Design, Assess, and Share framework for development of digital health technologies, which outlines the importance of ensuring that any significant changes in pathways are explored with service users [20].

The Unified Theory of Acceptance and Use of Technology (UTAUT) model suggests that there are several factors that influence individuals’ intention to use and actual use of new technologies [21]. This includes the extent to which a user believes that using the system will help them (performance expectancy), degree of ease of using the system (effort expectancy), influence of other people (social influence), and organizational and technical infrastructure to support the technology use (facilitating conditions). Therefore, before introducing a new technology to refine clinical pathways, it is important to evaluate the potential users’ acceptance and usability of the system (where usability is defined by 5 quality components: learnability, efficiency, memorability, errors, and satisfaction) [22].

This study aimed to explore acceptability from patient, caregiver, and HCP perspectives regarding home blood monitoring for patients with cancer who are receiving SACT.

## Methods

### Design

A qualitative study, using focus groups and semistructured interviews, was conducted.

### Participants

Patients were eligible to participate in the study if they were aged ≥16 years; had adequate English proficiency, without the need for an interpreter; and had received SACT within the past 12 months. Caregivers were eligible to participate if they were the caregiver of an eligible patient. HCPs were eligible if they were employed at the study site, and this group could include specialist registrars, fellows, consultants, nurses, and laboratory and information technology experts.

### Data Collection

Focus group and semistructured interviews were conducted over a 19-month time frame, between May 2019 and December 2020. Initially, focus groups were planned for all data collection, but the COVID-19 pandemic necessitated their immediate suspension. After March 23, 2020, only remote, semistructured, and one-to-one interviews were conducted with participants. Topics in the focus groups and interviews were identical and explored concepts of self-testing at home, expectations regarding responsibility for the device, and reporting the results. Participants used a prototype device to assess the ease of obtaining a capillary sample and inserting the cuvette into the device chamber. All interviews and focus groups were recorded, and notes were made during the sessions, ensuring that no identifiable information was included to maintain confidentiality.

### Procedure

Clinicians identified patients who were eligible to participate in the study, who were subsequently approached by the research team at their SACT administration visit or clinical review appointment. Before the COVID-19 pandemic, caregivers were approached at the same time as the eligible patients. During the pandemic, visitors to the clinical site were stopped, and therefore, no further caregivers were invited to participate after this time. Eligible HCPs from across the hospital were approached by the research team face to face, with details of the study provided in verbal and written form before agreement to participate.

Focus groups were formed based on participant availability, with patient and caregiver focus groups conducted separately from HCP focus groups. The focus groups had set objectives of areas that needed to be covered, as detailed in the topic guide (Multimedia Appendix 1), which informed the results. Once data saturation was achieved, no further focus groups or interviews were conducted.

### Ethics Approval

The study received ethics approval from the East of England – Cambridge South research ethics committee (reference 18/EE/0343; Integrated Research Application System project ID 234137). Every participant was given a participant information sheet and co-design booklet, with an opportunity for them to ask any questions before providing informed written consent.

### Data Analysis

Data collection and analysis occurred concurrently. Data collection ceased when data saturation was reached. Data were analyzed using a framework approach, guided by the UTAUT model. Framework analysis identifies commonalities and differences in the data, seeking to ascertain explanatory conclusions around themes [23]. This approach consists of 5 stages: familiarization, identifying a thematic framework, indexing, charting, and interpretation [24]. It is a highly systematic method of categorizing data using an inductive approach to generate themes [25]. All audio files were listened to and notes made during interviews and focus groups were read numerous times by 2 researchers (AV and ST) to familiarize themselves with the data. Key issues and concepts expressed by participants formed the basis of the thematic framework that was used to filter and classify the data. Transcripts were coded independently by AV and ST to create key themes. The themes were continuously reviewed to ensure the most accurate and concise representation of the data. Initial codes were cross-referenced with theory and previous studies. After creating a comprehensive code list, the final phase involved defining and naming themes. At this stage, both researchers reviewed the codes individually to decide which codes qualitatively described similar issues and therefore could be grouped together as an overarching theme. Both researchers created individual theme lists, and these lists were discussed to reach consensus. Following the creation of the framework, themes were indexed using the UTAUT model as a guide [21]. There were no significant differences in the data collected through interviews and focus groups across participant groups; therefore, a single thematic framework was created using all available data.

### Rigor

There are many different interpretations of what rigor means within qualitative research [26]. We have ensured that this study meets 3 key criteria, as discussed in the following sections.

### Reflexivity

Reflexivity is defined as the awareness of the researcher’s role within a study and how it is influenced by the focus of the study, thus enabling the researcher to acknowledge the way in which they affect the research process and outcomes [27]. Interviews were conducted by the device development team alongside researchers employed by the National Health Service trust who were not part of the patients’ clinical team. All of them have experience in interviewing people with cancer and conducted the interviews in an unbiased and balanced manner. Data analysis was conducted by 2 researchers (AV and ST), who used inductive approach and were unfamiliar with the relevant literature at that time. The researchers were not part of the device development team, which ensured transparency and quality, meaning that the data were analyzed without looking for preconceived ideas. The analysis included a process of self-critical reflection, acknowledging personal biases, preferences, and the research relationship.

### Credibility

Credibility ascertains whether the research findings are representative of the information obtained from participants’ original data and involve accurate interpretation of participants’ original views [28]. The researchers used a systematic approach to analyze the data, and a detailed explanation of the analysis stages is presented in this paper. Discussion between the 2 researchers tested the credibility and clarity of the analysis, to ensure that it reflected the participants’ experiences. Differences in interpretation were discussed, and agreement was reached.

### Transferability

Transferability describes not only behaviors and experiences but also their context and determines applicability to other settings [29]. The study provides useful findings, which can be directly translated into oncology clinical practice. The principle of these findings could also potentially be transferred to other clinical groups considering the use of remote monitoring techniques.

## Results

### Participant Characteristics

**Figure 1:**
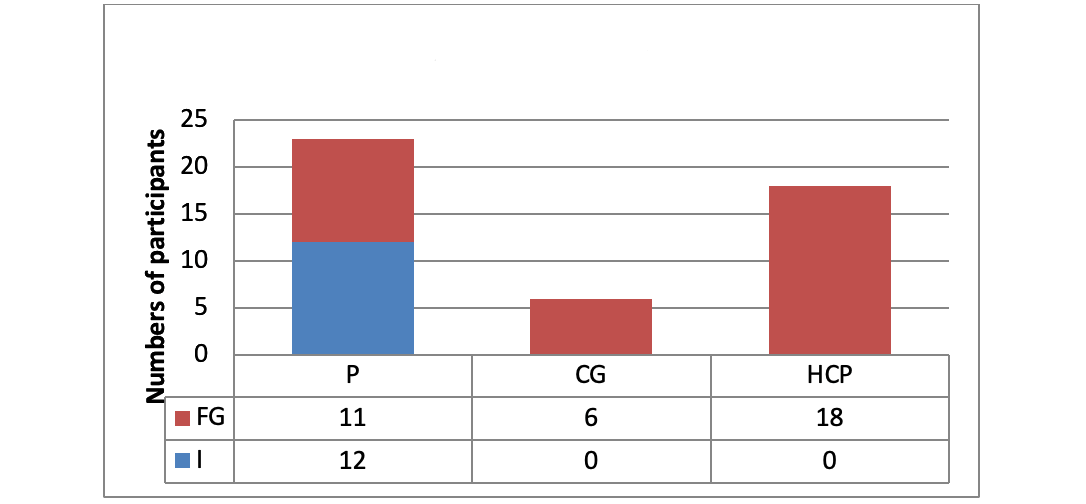
Participant breakdown. CG: caregiver; FG: focus groups; HCP: health care professional; I: interviews; P: patient.

Overall, 3 patient and caregiver focus groups, 2 HCP focus groups, and 12 semistructured interviews were conducted with 47 participants (Figure 1).

The 2 focus groups with HCPs were conducted before the implementation of COVID-19 restrictions. The first one, which was conducted on May 20, 2019, included a range of HCPs, both medical and nursing staff, and the second focus group, which was conducted on March 3, 2020, included SACT-trained nurses only. Of the 23 patient participants, most were women (n=19, 83%), with average age of 51 (SD 10.4; range 38-70) years. Of the 23 patient participants, 13 (57%) had breast cancer (Table 1).

**Table 1:** Patient characteristics (n=23).

Patient characteristics			Values
Age (years), mean (SD; range)			51 (10.4; 38-70)
**Sex, n (%)**			
	Male	4 (17)	
	Female	19 (83)	
**Disease group** **(cancer)** **, n (%)**			
	Breast	13 (57)	
	Ovarian	3 (13)	
	Colorectal	2 (9)	
	Prostate	1 (4)	
	Lung	1 (4)	
	Lymphoma	1 (4)	
	Upper gastrointestinal	1 (4)	
	Head and neck	1 (4)	

### Thematic Analysis

**Figure 2:**
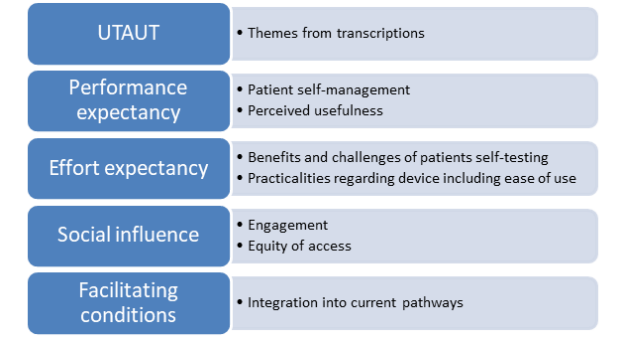
Themes. UTAUT: Unified Theory of Acceptance and Use of Technology.

In total, 4 overarching themes were identified, which aligned with the UTAUT model (Figure 2).

Quotations provided to support our analysis denote if the data were obtained from a patient, caregiver, or HCP focus group or patient interview, along with the date it was conducted (Table 2).

**Table 2:** Participant quotes to illustrate themes.

Themes and participant quotes		Identifier	Date
**Performance expectancy—patient self-management and perceived usefulness**			
	“I’d need a chart to interpret the results. There could be a high or low label and number, or a colour code. Like temperature checks; if it goes past a certain range you know to phone the hospital.”	PI^a^	November 2, 2020
	“I’d like to see the results, as I know my neutrophil count needs to be over 1 for my treatment to go ahead. Quick results would reduce my stress level.”	PI	November 9, 2020
	“I’m a nurse so I like to see my results as I understand what they mean. I run so I like to know how things are varying or stable. I monitor my results so that I know that I’m not being reckless to run.”	PI	November 11, 2020
	“It’s often given a lot of importance to the blood test but it’s about interpreting the results properly to avoid risks.”	HCPFG^b^	May 20, 2019
	“Anxious patients need to have it explained why they are being asked to do this kind of blood test; reducing the number of tasks required by the team and also the patient is key – support patient to interpretation. This is where the human factor is required.”	HCPFG	May 20, 2019
**Effort expectancy—benefits and challenges of self-testing**			
	“It will be good to regain some control by testing myself”	PFG^c^	June 17, 2019
	“To get to the hospital means I have to take two buses...it’s not an easy journey and COVID makes me not want to get public transport. It usually takes around 90 minutes to have my blood taken; waiting around isn’t nice and makes me anxious. Testing at home would be much better.”	PI	November 2, 2020
	“I hate needles, so a finger prick test would be better.”	PI	November 12, 2020
	“It can be really difficult to get my blood as my veins are hard to find. I wouldn’t sleep the night before; it stresses me massively...most stressful part of the treatment.”	PI	November 11, 2020
**Effort expectancy—practicalities regarding the device and ease of use**			
	“I’d value feedback that I’m doing it correctly.”	PI	November 4, 2020
	“It needs to be easy to use; if I can’t use it, I won’t use it.”	PI	November 2, 2020
	“Fewer steps will improve adherence.”	HCPFG	May 20, 2019
	“I would expect video format to get trained. Recording extra information will work well with the [NHS^d^ Trust] way of working.”	PFG	July 25, 2019
	“I’m not bothered what it looks like, as long as it works.”	PI	November 2, 2020
	“I wouldn’t have it out on display; it would remind me I am a patient. I would hide it in a cupboard. The smaller and nicer it looks the better.”	PI	November 11, 2020
	“The test would need to be clinician led – the patient should not be able to test whenever they want to.”	HCPFG	May 20, 2019
	“We would not want patients self-initiating tests because they could become obsessive...they could test out of hours when nobody is there to see it.”	HCPFG	March 3, 2020
	“If a patient hasn’t done their planned test or has and it is grossly out of range, there needs to be a safety netting system to ensure this isn’t missed.”	HCPFG	May 20, 2019
	“What happens if device doesn’t work – would treatment be delayed?”	PFG	June 17, 2019
	“A clear pathway is needed with a back-up solution if device fails.”	PFG	June 17, 2019
	“If the device doesn’t work the patient should be advised to contact the hotline for clinical advice, while the company resolves the issue with the device.”	HCPFG	May 20, 2019
**Social influence**			
	“I’d be concerned about elderly patients using the technology.”	HCPFG	May 10, 2019
	“What about those people who aren’t very tech savvy?”	HCPFG	May 10, 2019
	“Not everyone has internet at home, so would that mean they wouldn’t be able to use the device?”	HCPFG	May 10, 2019
	“Someone with poor dexterity may find [a test] tricky to do.”	PI	November 2, 2020
**Facilitating conditions—integration into current pathways**			
	“A visual or audio reminder on the device about to take the test would be helpful.”	PFG	July 25, 2019
	“Being notified when the results have been seen would be really helpful.”	PFG	July 25, 2019
	“It’s really important patients receive confirmation that a test has been received.”	HCPFG	May 20, 2019
	“What happens if it’s a ‘bad’ result and no face to face option? That would make me very anxious.”	PFG	June 17, 2019
	“There needs to be time within the [clinician’s] job plan that is allocated to reviewing bloods taken on the device in order to ensure that results are reviewed in a timely manner.”	HCPFG	May 20, 2019
	“It is crucial to ensure results are not lost and are all reported on one system.”	HCPFG	May 20, 2019
	“Real time transmission of information is needed that can update all the scheduling systems to avoid future delays and issues.”	HCPFG	March 3, 2020

^a^PI: patient interview.

^b^HCPFG: health care professional focus group.

^c^PFG: patient focus group.

^d^NHS: National Health Service.

### Performance Expectancy—Patient Self-management and Perceived Usefulness

Performance expectancy relates to the degree to which a user believes that using the device will assist them to attain a perceived gain [30].

Our findings illustrate contrasting opinions from patients regarding what information is shared on the device and how and what level of autonomy is expected and desired in terms of interpreting that information. Some patients were very keen to see their blood results and felt that they could interpret what they meant, which would inform the self-management aspect of their care. However, other patients felt that knowing their blood test results would make them anxious, particularly if their results were not within the desired parameters to proceed with treatment. HCPs also expressed concern about patients being able to see their results and the associated anxiety regarding their interpretation. There was consensus that patients could see results, but strict guidance is needed to support patients’ interpretation.

### Effort Expectancy

Effort expectancy relates to the amount of perceived effort required to use a new technology, which, in this study, is a home blood monitoring device. If the device is perceived to be easy to use, it is more likely to be adopted [31].

#### Benefits and Challenges of Patient Self-testing

In our study, all patient participants (23/23, 100%) were happy with the concept of self-testing and could see how it would be beneficial to them. Patients highlighted that a reduction in time spent at the hospital and the reduced need for a venous blood test would be highly beneficial and welcomed, because several patients found having their blood taken as stressful. However, 22% (5/23) of the patients highlighted the potential for self-testing to increase anxiety if they could not obtain feedback about their results or discuss them with a clinician.

#### Practicalities Regarding the Device and Ease of Use

Regarding effort expectancy, participants stressed the importance of appropriate training and feedback to ensure that the device was being used correctly. Having appropriate feedback and training would greatly influence the patients’ ease of use and confidence in the device. A participant supported home testing but felt more comfortable with communicating in person. Both patients and HCPs identified the need for simplicity of the testing process to improve engagement.

Video training was discussed in a focus group, and all participants (4/4, 100% of the patients and 2/2, 100% of the caregivers) agreed this would be useful. Most participants (32/47, 68%) were not concerned about the appearance of the device if they had the trust that it works effectively. Others highlighted that they would want to store it out of sight, so that they were not reminded of their status as a patient.

HCPs discussed the timing of testing. They felt that patients should only conduct a test when it was clinically required; it was suggested that the device should be locked remotely, with the patient only able to perform a test at a scheduled time. Their concerns related to patient safety, in terms of ensuring that patients’ test results were not ignored or “lost” in the system. Both patients and HCPs expressed concerns regarding potential device failures and questioned what would happen in this instance.

### Social Influence—Engagement and Equity of Access

Social influence refers to a belief about technology acceptance and use based on the influence of significant others, such as family and friends, whom they respect [32]. Social influence significantly affects the individual’s intention to use home blood monitoring.

Patients and HCPs were concerned about equity of access, as they felt that not everyone may be able to engage for various reasons, with reference to those who had little or no support at home being less likely to conduct home blood testing. Concerns were raised, which were on a practical and physical level, highlighting issues around dexterity, digital naivety and inexperience, and internet access, all of which could impede the uptake of home blood monitoring.

### Facilitating Conditions—Integrating Into Current Pathways

Facilitating conditions is defined as the degree to which a user believes that an organizational and technical infrastructure exists to support the use of this technology [33].

Patients, caregivers, and clinicians discussed the logistics of how home blood monitoring would fit within the current care pathways and existing technical infrastructure. There are several components in this theme: communication between the patient and their clinical team regarding the test, practicalities of presenting the test results to the patient, and the digital interface between the device and hospital systems. Patients and caregivers were more focused on how the test would be scheduled, recorded, and reported, with particular emphasis on whether the test results will be displayed on the device. HCPs expressed the importance of allocating time within their job plans to ensure that the results are reviewed in a timely manner. All the parties highlighted that acknowledgment of receipt of results by the clinical team would be beneficial. Several HCPs (6/18, 33%) suggested that the device should be integrated into existing test reporting systems, rather than adding to what is already a complex landscape of digital technology systems.

## Discussion

### Principal Findings

This study explored patient, caregiver, and HCP perceptions about home blood monitoring for patients receiving SACT to guide prototype development and refine clinical pathways. A total of 47 participants participated in the interviews or focus groups. Framework analysis of transcripts was conducted using the UTAUT model to underpin the 4 themes that were identified.

The concept of home FBC monitoring has been under development for several years, with the goal of transforming the delivery of cancer care in a way that suits the needs of patients, while minimizing the burden on health care systems. Home blood monitoring has the potential to redefine cancer care, positively affecting the experience of patients and their caregivers, and improve the efficiency of health care resources. In addition to being easy to use, technological interventions should positively affect disease management and quality of life [26]. The UTAUT model highlights the different elements that influence whether and how an individual will use a technology. In this study, we found that participants felt that there were significant benefits of home blood monitoring, such as giving them more control across their cancer journey and reducing the burden associated with hospital appointments. This aligns with performance expectancy, which refers to how helpful a digital intervention is perceived to be by the user, which will influence intention for use [21]. In other diseases, such as asthma, chronic obstructive pulmonary disease, and diabetes, remote monitoring coupled with education and support from the clinical team has been proven to reduce health care use, improve patients’ quality of life, and support patients to self-monitor and manage their condition more efficiently [34-36]. Participants in our study highlighted that many medical reviews had moved to remote consultations during the pandemic. This was positively accepted as it saved time and the associated travel costs in comparison with face-to-face appointments. Home blood monitoring coupled with remote clinical consultations would maximize the concept of remote monitoring, thus enabling a more thorough and robust assessment. HCPs felt that a test should be initiated by a clinician, rather than being patient-led, but some patients felt that they would want to test whenever they felt unwell. Other studies have found that self-tests are frequently performed owing to curiosity from the patient, to gain reassurance [37]. Self-testing can make patients feel empowered, but there is evidence that a danger of *lay ignorance* exists in conducting a test that is unlikely to provide the answer they are looking for [38]. Contrasting studies have found that some participants did not want to be burdened with self-testing owing to additional stress and negative connotations of sickness from having equipment at home [39]. This emphasizes the need for an opt-out option for patients, to minimize any feelings of coercion or discrimination.

A crucial predictor of technology acceptance relates to effort expectancy, and there is evidence suggesting that easy access to technical advice with low effort burden increases use [40]. Ensuring that patients feel confident and competent in using any home monitoring system is key to its effectiveness. If a digital intervention is usable, it promotes engagement, productivity, efficiency, and pleasure in use [22]. Patients raised concerns regarding their ability to perform the test correctly and questioned the availability of training and support. Usability refers not only to *ease of use* but also to infrequent and regular use, to enable users to achieve their goals, which, in this case, is to obtain an accurate FBC result [41]. A robust education program will improve the degree of ease associated with use of the device. Dexterity and sensory dysfunction were other concerns raised as potential barriers to the uptake of home blood monitoring. Of the 47 participants, 2 (4%) participants reported chemotherapy-induced peripheral neuropathy. Although this did not affect their dexterity, it may be an issue for some patients and needs to be considered and explored with potential users. Similarly, diabetes can cause sensory and visual disturbances, and a comparative study regarding the accessibility of blood glucose monitoring for people who are blind and visually impaired recommended that modifications needed to be made to the blood glucose monitors to make them equitable and safe for all [42].

Social influence was the third theme and is a significant factor in the use of a new digital intervention, with encouragement from surrounding people being a motivational factor [43]. Reticence to engage in remote monitoring may be because of feelings of isolation owing to loss of physical contact and face-to-face communication with the clinical team. Remote monitoring needs to be embraced by clinicians to ensure that patient communication, education, and support are not compromised and that strong patient-clinician relationships are maintained. It is important to communicate that not all patient care will be delivered remotely; there will always be a need for face-to-face consultations and further venous sampling. This corresponds with previous studies exploring remote monitoring, which found that including features facilitating patient-clinician interaction may encourage engagement [44]. Although all our participants (47/47, 100) had access to the internet, they raised concerns related to individuals who may not have that access. A cross-sectional study of 151 patients with cancer exploring barriers to and enablers of patients’ current and desired uptake of health care technology found that more than one-fourth of their sample did not have daily access to the internet and approximately one-third did not own a smartphone capable of displaying mobile apps, with age being a factor [45]. The oldest participant in our study was aged 70 years (mean age of the sample was 51, SD 10.4 years). With an aging population, it would be beneficial to conduct purposive sampling to gain feedback from those aged >70 years to ensure that their input is used in the design process, as the strength between perceived usefulness and intention to use varies with age [46].

The final theme was facilitating conditions. This is a crucial predictor of technology acceptance [47]. The creation of guidelines and their integration into existing clinical pathways with review procedures are critical. If not addressed, common usability problems can affect adoption [21]. Adopting a theory-driven approach will increase buy-in and trust, thus improving effectiveness and scalability [34]. The decision to adopt a digital intervention is complex, with attitudinal, social, and environmental factors having an impact [48]. The UTAUT model acknowledges that broad contextual factors may facilitate or inhibit digital adoption [21]. This supports our study findings, which identified the importance of digital interoperability between systems to promote usability, reassurance, and communication. As emphasized by patient participants in our study, there was concern regarding how they would know whether their blood test result had been seen, and similarly, HCPs wanted to ensure that they had protected time to review the tests to promote patient safety and increase confidence in the pathway. In similar studies that have evaluated remote monitoring in diabetes, potential safety concerns were highlighted if clinicians were unable to review the results in a timely manner [37]. Increase in cognitive burden for clinicians is a known barrier to the adoption of digital interventions [49]. A crucial factor, raised by all stakeholders, was the need to ensure that there are safety measures implemented in case of device failure or lack of patient engagement. There should be an alternative solution to ensure that cancer care can still be delivered safely and equitably. To maximize the potential benefit from new innovations, guiding principles should involve transparency, equitable access, ethics, ownership, and sustainability [35]. Risk of harm and compromised privacy of users is unacceptable and will detrimentally affect acceptance and use [50]. In contrast, the more disabled a person is, the more willing they may be to accept technology that reduces privacy if there is a belief that the intervention will improve independence and quality of life [51].

### Limitations

This study recruited participants from a single site in the National Health Service in the United Kingdom; therefore, the findings may not be generalizable to other cancer centers or health care settings, and further studies would be required to determine this. Focus groups and interviews were conducted over a prolonged period, with the COVID-19 pandemic having implications upon recruitment. Participants were purposively selected following insight from the clinical teams, meaning that those who would be most likely to engage were approached. An assumption can be made that by agreeing to participate, participants were already open to the concept of home blood monitoring.

### Conclusions

The development of minimally disruptive health care strategies for people living with cancer who are receiving SACT is essential to optimize the quality of life, while simultaneously improving the efficiency of hospital resources. This study highlights patient, caregiver, and HCP acceptance of the concept of home blood monitoring. Several chronic conditions already use home monitoring and demonstrate clear benefits in terms of patient experience and disease management. To invest in medical devices and implement changes to existing clinical pathways, there must be evidence that this is financially viable and clinically beneficial. For patients with cancer receiving SACT, home blood monitoring could expedite FBC results and thus provide more timely access to evidence-based care. Feedback obtained in this study has demonstrated eager acceptance from all stakeholders. However, although they are often considered synonymously, self-testing and self-management are not mutually exclusive, and this study illustrated some disparity in opinions regarding patient self-management. Further studies are needed to determine how and where home blood monitoring would fit within clinical pathways, in a way that is robust and equitable.

## Appendix

Multimedia Appendix 1 Topic guide.

## Abbreviations

FBC: full blood count
HCP: health care professional
SACT: systemic anticancer therapy
UTAUT: Unified Theory of Acceptance and Use of Technology
