# Supplementary material for: Assessing the Acceptability of Home Blood Monitoring for Patients With Cancer Who Are Receiving Systemic Anticancer Therapy From a Patient, Caregiver, and Clinician Perspective: Focus Group and Interview Study
Source: JMIR Nurs. 2023 Jan 6;6:e39815. doi: 10.2196/39815 (PMC9862331; doi:10.2196/39815)
Supplement: Multimedia Appendix 1 [file nursing_v6i1e39815_app1.docx]

**Study title:** PERTH 2: Co- design of a home-based monitoring service for

cancer patients, carers and healthcare professionals to optimize delivery of systemic cancer therapy

**IRAS ID:** 234137

**REC ref:**  18/EE/0343

### **Topic guide for focus groups and 1 to 1 interviews**

**The questions, activities and topics below will be used in one to one interviews and focus groups.**

**Photos, audio recording, video - these may be taken if the interviewee/s agree, these will be used only by the Sponsor team to document personas of the users, part of Sponsor’s Quality Assurance process.**

Topics and sample questions relating to cancer and personal health:

How do you communicate about your health status/treatment with your healthcare provider?

How do you track and identify side effects and symptoms and communicate them with your healthcare provider/relatives/colleagues?

How often do you check in with your doctor during treatment?

Who do you contact/think you need to see when you are feeling ill or worried?

Who do you contact in an emergency and non-emergency situation?

Can you describe a scenario when you have been ill at home?

How do you decide to go to A&E?

Do you understand your results and can you interpret them?

Do you feel that you need to see a healthcare provider every time you feel ill?

What are your feelings about home-care monitoring and testing at home?

How comfortable are you with fingerpricking?

Can you describe your visit to the doctor or hospital?

(prompt: making an appointment, is blood test always done? Is blood test done at the same time as doctors appointment, waiting time for results, follow up with doctor)

Where do you see a home monitoring device used during cancer care fitting in with other products you may have i.e. would you consider it more similar to a activity tracker, a blood pressure monitor or a glucose monitor?

What is important for you to trust a home monitoring device or brand?

Do you use any other measuring or tracking devices like scales, blood pressure monitor, cholesterol tests or glucose monitors, sleep tracker, activity tracker, thermometers, cancer related apps?

How do you review, triage and act on data received from a home monitoring device?

How do you manage third party device data from third party devices in your daily routine?

Who takes responsibility of data from third party services?

How could patient home monitoring make your workflow more efficient?

Is the use of self-care encouraged?

Exploring delays to treatment. How do you trace, document, and make decisions made from delays?

How is treatment re-scheduled? (toxicity assessment, symptoms and blood tests etc)

**Activity:**

Card sorting activity. These cards will have features that Sponsor have decided could be a part of the service.

The participant/s is asked to sort cards into columns and into order of importance. For example, three columns entitled ‘I wouldn’t use this unless it….’ ‘It would be nice if this could….’ ‘I don’t see the importance of…’

The interviewer will discuss the cards with the participant/s to gain an understanding of their logic.

Cards have wording such as:

Tracking trends in my blood results over time

Tracking my diet

Tracking my treatment (e.g. pills)

Seeing the effect of different treatments.

Correlating and hb over time

Symptoms tracking (nausea, etc)

Being able to show my boss that my blood results are low/is very low

Being able to share my blood results with my family and friends

Sharing my blood results and experience with support groups

Automatically sending my device results to my doctor

No more doctor visits

More doctor visits

Fewer doctor visits

Documenting my result to show my doctor when I have an appointment

Ordering my prescription

Access to an online community where I could talk about cancer, share experiences, and ask questions

Proof for my health insurance

Access to a support helpline

Blank cards and pen for people to add in their own ideas will also be provided.

#### **Other Activities may include:**

Creating sketch prototypes

Ideas into drawings, scenarios, and models to create design proposals

Assessing small variations of the design.

Testing high-quality on-screen prototypes and answering questions to help with further refinement of the prototypes

Role playing

Ease of use the test strip for blood collection

Making models

Sketching

Branding exercise

Creating and assessing mood boards

Brainstorming

Reviewing instructions and tips to use the service

Reviewing marketing and educational material

UI review

Evaluate written material at home

Assess take home materials

Complete short exercises at home e.g filling in simple symptom tracking sheet

Assessment of the service to include the app and digital plaftorm

## Topics and sample questions relating to the usability of the device:

1. Did you have all the information you needed to use the service/device?
2. Did you have all the equipment you needed to use the service/device?
3. Does the device do everything you want it to do?
4. Is the service meeting your expectation?
5. How many measurements do you want the device to run?
6. Please assess the different subscription models presented for consumables.
7. Was the user interface easy to use?
8. What did you find difficult about the user interface?
9. What improvements could be made for the user interface?
10. Was the test disposable easy to use?
11. Do you think you will need a lot of practice to use the test disposable properly?
12. Was the lancet easy to use?
13. Did using the lancet hurt?
14. How would you improve the lancet?
15. Do you prefer finger pricking or blood being drawn from veins?
16. Are the results easy to understand?
17. Are the results presented in a way that makes them useful?
18. Is it useful to link the device results to other data such as diet, exercise, sleep etc?
19. Do you share the results of blood tests?
20. Would you be happy to share your health data for research?
21. Is the service convenient?
22. Does the device fit well with your lifestyle?
23. Have you identified any advantages or disadvantages in using a home-based device to monitor blood counts during cancer treatment?
24. Consumable easy to use, store and dispose of

### Table of topics for discussion

| Elements to assess.  Criteria | Device (include display) | Test disposable | Holder | Charger | Lancet | Packaging | IFU | UI | Leaflet | App | Website | Social Networks | Technical Support |
| --- | --- | --- | --- | --- | --- | --- | --- | --- | --- | --- | --- | --- | --- |
| Robustness | x | x | x | x |  | x |  |  | x |  |  |  |  |
| Usability | x | x | x | x | x | x | x | x | x | x | x | x |  |
| Self-explanatory | x | x | x | x | x |  |  |  |  |  |  |  |  |
| Visible language * | x |  |  |  |  |  | x | x | x | x | x | x |  |
| Responsivity | x |  |  |  |  |  |  | x |  | x | x | x | x |
| Connectivity | x |  |  |  |  |  |  |  |  | x | x |  |  |
| Compatibility | x |  |  |  |  |  |  |  |  | x |  |  |  |
| Battery lifetime | x |  |  |  |  |  |  |  |  |  |  |  |  |
| Display quality | x |  |  |  |  |  |  |  |  |  |  |  |  |
| Brightness /contrast | x |  |  |  |  |  |  |  |  |  |  |  |  |
| Navigability |  |  |  |  |  |  |  | x |  | x | x |  |  |
| Visual hierarchy |  |  |  |  |  |  | x | x | x | x | x |  |  |
| Text size |  |  |  |  |  |  | x | x | x | x | x |  |  |
| Button size | x |  |  |  |  |  |  | x | x | x | x |  |  |
| Button shape | x |  |  |  |  |  |  | x |  | x | x |  |  |
| User feedbacks  (messages, sounds) | x |  |  |  |  |  |  | x |  | x |  |  |  |
| Relevant content |  |  |  |  |  |  |  |  |  | x | x | x |  |
| Helpful content |  |  |  |  |  |  | x | x | x | x | x | x |  |
| Entertaining content |  |  |  |  |  |  |  |  |  |  |  | x |  |
| Credibility |  |  |  |  |  |  |  |  |  | x | x | x |  |
| Reminders/ notifications | x |  |  |  |  |  |  | x |  | x |  |  |  |
| Design | x | x | x | x | x | x | x | x | x | x | x | x | x |

*(simplicity, clarity, distinctiveness, emphasis)

Example of questions that can be derived from this table:

- on a scale from 1 to 5 , how responsive is the UI?
- If UI is not easy to navigate, what element would you add/rearrange/delete?
- Can you point at chapters that are not helpful enough in the IFU?
- Do you find the way written and visual information are displayed In the IFU helpful?
- Why is the packaging not robust enough?
- Do you find the feedbacks given by the device useful? Would like to have sound feedbacks?
- Was it easy to understand how to use the lancet?
- Can you list topics you are interested on for our web publication?
- Did you manage to navigate easily in the UI, is it intuitive? Can you point any source of frustration or restriction during while navigating?
- Are you satisfied with the display quality and responsiveness? Especially accessibility of button features and text size.
- On a scale 1 to 10, how easy was it to use the device combined with the holder and the test disposable?
